# Author Correction: Learning single-cell perturbation responses using neural optimal transport

**DOI:** 10.1038/s41592-023-02073-w

**Published:** 2023-10-30

**Authors:** Charlotte Bunne, Stefan G. Stark, Gabriele Gut, Jacobo Sarabia del Castillo, Mitch Levesque, Kjong-Van Lehmann, Lucas Pelkmans, Andreas Krause, Gunnar Rätsch

**Affiliations:** 1 Department of Computer Science, ETH Zurich, Zürich, Switzerland; 2 AI Center, ETH Zurich, Zürich, Switzerland; 3 Medical Informatics Unit, University of Zurich Hospital, Zürich, Switzerland; 4 Swiss Institute of Bioinformatics, Zurich, Switzerland; 5 Department of Molecular Life Sciences, University of Zurich, Zürich, Switzerland; 6 Department of Dermatology, University of Zurich Hospital, University of Zurich, Zürich, Switzerland; 7 Cancer Research Center Cologne–Essen, Site: Center Integrated Oncology Aachen, Aachen, Germany; 8 Department of Biology, ETH Zurich, Zürich, Switzerland

**Keywords:** Computational biology and bioinformatics, Machine learning, Molecular biology

Correction to: *Nature Methods* 10.1038/s41592-023-01969-x, published online 28 September 2023.

In the version of this article initially published, the online Methods were missing and are now restored in the HTML and PDF versions of the article.

