# Supplementary material for: Learning single-cell perturbation responses using neural optimal transport
Source: Nat Methods. 2023 Sep 28;20(11):1759–68. doi: 10.1038/s41592-023-01969-x (PMC10630137; doi:10.1038/s41592-023-01969-x)
Supplement: Supplementary file 1 — Summary of related work, Supplementary Figs. 1–13, Table 1, Materials, Experimental details, and References. [file 41592_2023_1969_MOESM1_ESM.pdf]

nature methods

Article https://doi.org/10.1038/s41592-023-01969-x

Learning single-cell perturbation responses using neural optimal transport

In the format provided by the authors and unedited

SUPPLEMENTARY INFORMATION

Learning single-cell perturbation responses using neural optimal transport

Charlotte Bunne,[1,2,*] Stefan G. Stark,[1,2,3,4,*] Gabriele Gut,[5,*]
Jacobo Sarabia del Castillo,[5] Mitch Levesque,[6] Kjong-Van Lehmann,[1,7]
Lucas Pelkmans,[5,†] Andreas Krause,[1,2,†] Gunnar Rätsch[1,2,3,4,8,†]

[1] Department of Computer Science, ETH Zurich, Switzerland;
[2] AI Center, ETH Zurich, Switzerland;
[3] Medical Informatics Unit, University of Zurich Hospital, Switzerland;
[4] Swiss Institute of Bioinformatics, Switzerland;
[5] Department of Molecular Life Sciences, University of Zurich, Switzerland;
[6] Department of Dermatology, University of Zurich Hospital, University of Zurich, Switzerland;
[7] Cancer Research Center Cologne-Essen, Site: Center Integrated Oncology Aachen, Germany;
[8] Department of Biology, ETH Zurich, Switzerland.

*These authors contributed equally.
†To whom correspondence should be addressed: kjlehmann@ukaachen.de, lucas.pelkmans@mls.uzh.ch, krausea@ethz.ch, gunnar.raetsch@inf.ethz.ch.

A Related work **2**
A.1 Single-cell perturbation response prediction 2
A.1.1 Modeling perturbation responses as shifts in latent space 2
A.1.2 Modeling perturbation responses via matching of subpopulations 3
A.2 Single-cell perturbation response analysis 4
A.3 Optimal transport in single-cell biology 4

B Additional results **5**

C Materials **13**

D Experimental details **15**
D.1 In-vitro experiments 15
D.2 In-silico experiments 15

A Related work

In the following, we provide an overview of methods predicting population responses and recent developments on optimal transport for single-cell biology data.

A.1 Single-cell perturbation response prediction

With increasing data availability, a diverse set of approaches has been proposed to model cellular perturbation responses, ranging from mechanistic to current deep learning-based approaches. Mechanistic models (82, 83) define mathematical models of molecular interactions to model the effect of perturbation. These methods, however, are restricted to simpler and well-understood systems as they do not capture highly nonlinear perturbation responses of a heterogeneous cell population. Further, these methods are limited in their applicability as they do not scale to genome-wide measurements (84, 85, 86). Linear models (87, 88), on the other hand, predict changes in cellular gene expression levels using regularized regression methods, where the model predicts a gene's expression level as a linear combination of effects of different perturbations, fitting the regulatory effect of each perturbation on each gene. Due to assuming only linear relationships of individual genes in response to a perturbation, these methods are similarly unable to capture complex and inhomogeneous population responses upon perturbation. Heydari et al. (89), on the other hand, predict perturbation responses through inferring the underlying gene regulatory network. Prediction of the perturbed states is achieved through a dynamic simulation of those logical gene networks. Thus, the predicted perturbed states are restricted to only the selected set of genes used to build the corresponding regulatory network. Lastly, current state-of-the-art methods (90, 91, 14) aim to learn low-dimensional representations of inputs using autoencoders such that perturbation effects can be applied with simple linear interpolations in representation space. Thus, they predict perturbation responses via linear shifts in a learned low-dimensional latent space. These models are attractive because they are fully parameterized, enabling us to make predictions on unseen cells. By tackling the task of perturbation response predictions via the even more challenging task of learning a meaningful low-dimensional embedding, these methods can be expected to, at best, only perform moderately well. Therefore, we sought to learn a fully parameterized perturbation model that robustly describes the cellular dynamics upon intervention while accounting for underlying variability across samples. More details on both methods are provided in Supplementary Section A.1.1.

A.1.1 Modeling perturbation responses as shifts in latent space

Consider a single-cell dataset of a binary perturbation. Let $\{x_1 \dots x_n\}$, $x_i \in \mathcal{X}$, drawn from $\rho_c \cup \rho_k$ and let $c(i) \in \{0, 1\}$ indicate the perturbation status of a single cell,

$$c(i) = \begin{cases} 0, & \text{if } x_i \sim \rho_c \\ 1, & \text{if } x_i \sim \rho_k. \end{cases}$$

scGen Given representations $\{z_1 \dots z_n\}$ of $\{x_1 \dots x_n\}$, learned by an autoencoder, with encoder ϕ and decoder ψ, scGen (91) predicts a perturbation response using latent space arithmetic. Let $\bar{z}^{(l)}$ be the mean of representations in condition l

$$\bar{z}^{(l)} = \frac{1}{|\{i : c(i) = l\}|} \sum z_i \delta_{c(i)l},$$

the perturbed state of $x' \sim \rho_c$ is predicted as

$$\psi(\phi(x') - \bar{z}^{(0)} + \bar{z}^{(1)}).$$

cAE The conditional autoencoder is based on a popular batch correction technique within the single-cell community, first introduced by (90). It introduces condition-specific parameters into the encoder and decoder that attempt to remove and replace information in the data

specific to their conditions. They operate by concatenating one-hot encodings of condition labels (here, perturbation status) to the inputs of the encoder and decoder. These encodings, in effect, make the bias term in the first layer of the encoder and decoder a learnable parameter specific to each condition. I can thus be considered equivalent to learning a linear shift in the latent space. Given an encoder ϕ and decoder ψ, the network is trained to reconstruct cells conditioning on its true label

$$z_i = \phi(x_i|c(i)), \qquad \hat{x}_i = \psi(z_i|c(i)).$$

Once trained, the perturbed state of $x' \sim \rho_c$ is predicted as

$$z_i = \phi(x'|0), \qquad \hat{x}' = \psi(z_i|1).$$

A.1.2 Modeling perturbation responses via matching of subpopulations

Within one sample distinct cell types might exhibit very different responses toward a perturbation. This heterogeneity suggests modeling perturbation effects by first identifying different subpopulations and then predicting the response for each of those subpopulations individually. In the following, we introduce a method built upon that insight, which serves as a baseline in this study.

Chen et al. (92) predict gene expression changes that occur in complex single-cell populations by identifying distinct subpopulations within that heterogeneous mixture for both the control ρ_c and treated population ρ_k, and comparing as well as aligning those subpopulations in control and treated state through a probabilistic model. To robustly identify those subpopulations, the data $X = \{\mathbf{g}_i\}_{i=1}^k$ consisting of n-dimensional gene vectors $\mathbf{g}_i = (g_i^1, g_i^2, \dots, g_i^n)$ for each of the k cells is embedded in a lower m-dimensional space using orthogonal nonnegative matrix factorization (oNMF) (93), i.e., $Z = \{\mathbf{c}_i\}_{i=1}^k$ with $\mathbf{c}_i = (c_i^1, c_i^2, \dots, c_i^m)$. oNMF is known to produce a meaningful set of features as the resulting representation is a superposition of largely disjoint features, here genes, shown to work well for clustering tasks. This cluster structure then serves as the foundation to identify and represent subpopulations in both the control and subpopulation as l independent Gaussian mixtures, i.e., $P(Z) = \sum_i^l w_i \mathcal{N}(Z; \mu_i, \Sigma_i)$ with weights w_i, centroids μ_i, and covariance matrices Σ_i. These parameters associated with each Gaussian density (w_i, μ_i, Σ_i) have a natural correspondence to the biological structure and semantics of those cell populations. Lastly, to understand the perturbation response of each subpopulation, Chen et al. (92) align subpopulations identified in the control population to those identified in the target population based on a measure of *closeness*, such as Jeffrey's divergence utilized in their work. The resulting statistical alignment allows us to determine the subpopulation-specific perturbation effect through the change in all parameters, i.e.,

$$\begin{aligned}
\Delta\boldsymbol{\mu}_i &= \left\|\mu_i^{\text{control}} - \mu_j^{\text{treated}}\right\|_2, \\
\Delta\boldsymbol{\Sigma}_i &= D_{\text{C}}\left(\Sigma_i^{\text{control}}, \Sigma_j^{\text{treated}}\right), \\
\Delta w_i &= \left|w_i^{\text{control}} - w_j^{\text{treated}}\right|,
\end{aligned}$$

with D_C denoting the Forstner metric. Predictions on unseen control cells can be thus obtained by projecting each cell into the low-dimensional oNMF space, assigning each cell to the corresponding subpopulation, obtaining the corresponding treated state through modeling the perturbation effect via $(\Delta\mu, \Delta\Sigma, \Delta w)$, and lastly, projecting these predicted treated cell states back into the original n-dimensional gene space.

In order to utilize PopAlign as a baseline, we follow the implementation and hyperparameter choices suggested by Chen et al. (92).

A.2 Single-cell perturbation response analysis

Beyond these tools, a series of methods have been developed to study the nature of perturbation effects on single-cell data. Several works hereby have concentrated on deciphering and disentangling various cellular and genetic patterns within perturbation responses. Chen et al. (94), for example, provide a tool for uncovering different axes of cell variation. A pairwise comparison of identified cell subtypes thereby allows an analysis of patient-to-patient variability. Similarly, Bhalla et al. (95) derive a patient similarity network that identifies patient subgroups by analyzing genetic and molecular landscapes from multi-omics data. Instead of clustering patients with similar perturbation responses, other tools have tried to dissect variability on the cell level. For this, Chari et al. (96) cluster PCA-based representation of control and perturbed cell populations. Given cell type annotations, they quantify perturbation effects by computing the ℓ_1 distances between centroids of each cell type cluster. Skinnider et al. (97) construct a classifier-based framework where cell types most responsive to perturbations show a high separability between control and treated cell states within a high-dimensional space. Burkhardt et al. (98) achieve a similar analysis by introducing a continuous measure based on the relative likelihood estimate of observing a cell in each experimental condition. Lastly, Petukhov et al. (99) provide a computational suite to carry out statistical tests that, among others, allows to test variability between different samples and conditions. Lastly, due to the absence of ground truth when predicting single-cell perturbation responses, various methods have concentrated on simulating single-cell RNA-seq data that capture important properties of experimental data. Cao et al. (100) provide a comprehensive benchmark study for simulation methods, while at the same time introducing evaluation metrics to measure quantitative and qualitative properties of the RNA-seq data generated by various methods. Most importantly, while all those methods contribute to a better understanding of single-cell perturbation responses, they do not allow to predict perturbed states of unseen unperturbed cells, such as those from an incoming patient.

A.3 Optimal transport in single-cell biology

Following pioneering work by Schiebinger et al. (101), numerous problems in single-cell biology have recently been approached using optimal transport. These problems include mapping cells across perturbations, time points, experimental batches, as well as reconstructing spatial structure from gene expression. In contrast to previous approaches (101, 102), we seek to learn and thus parameterize the optimal transport map T_k to allow forecasting and predictions on *unseen* cell populations, i.e., in the out-of-sample setting. Existing methods addressed proposed neural network-based OT models that directly parameterize T_k (103, 104, 105). This, however, has been shown to yield an unstable and difficult-to-solve optimization problem (106, Table 1). In this work, we take a different path: Instead of parameterizing the optimal transport map T_k, we follow Makkuva et al. (106) and parameterize the convex potentials of the dual optimal transport problem f and g by convex neural networks (107). Brenier's theorem (59) allows us to recover the optimal map T_k using the gradient of a convex function g_k, i.e., ∇g_k. Enforcing the spatial regularity of the pushforward map T_k using optimal transport as modeling prior and parameterizing a pair of dual potentials yield a tractable learning problem and are central to the success of CellOT.

B Additional results

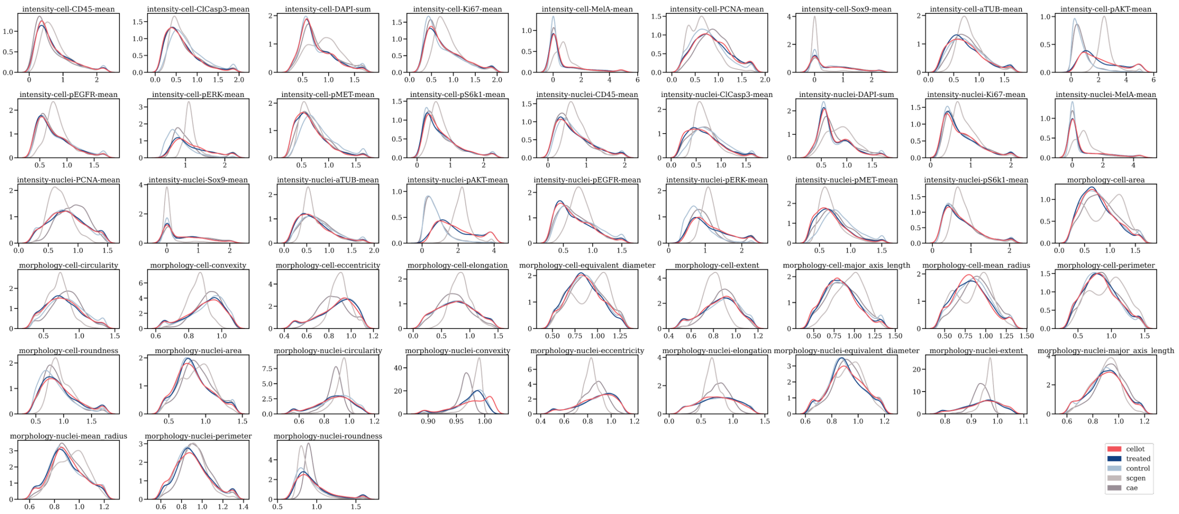

Supplementary Figure 1: Predicted and observed marginals of cells profiled by 4i, treated with Imatinib. All extracted intensity and morphology features are shown.

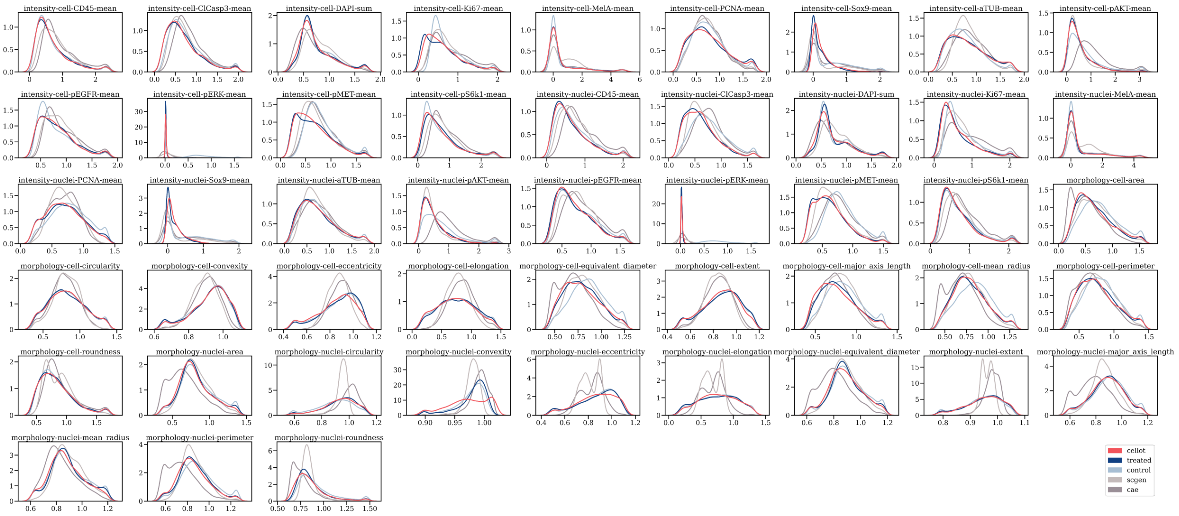

Supplementary Figure 2: Predicted and observed marginals of cells profiled by 4i treated with Trametinib. All extracted intensity and morphology features are shown.

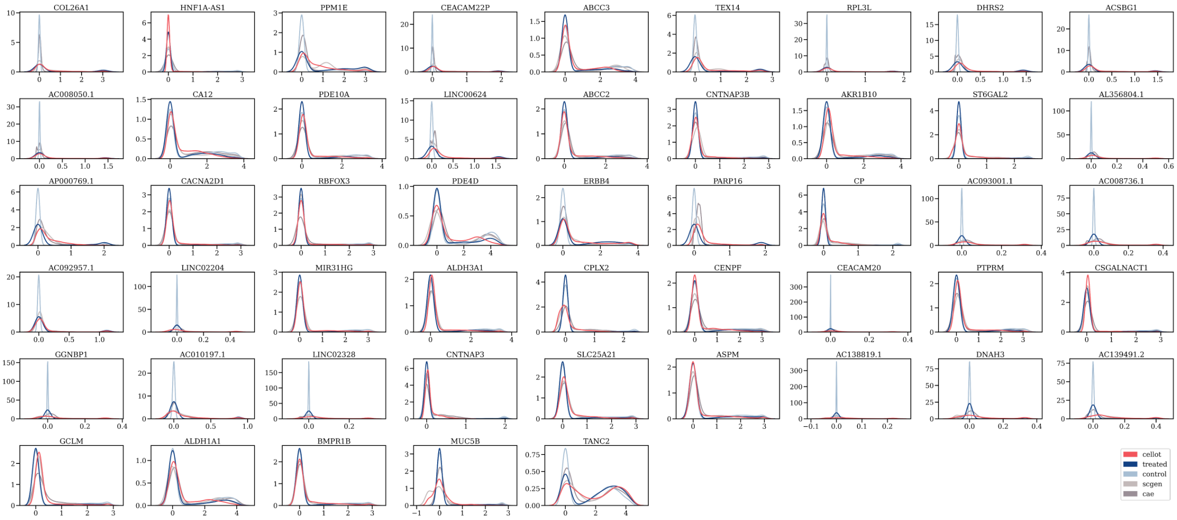

Supplementary Figure 3: Predicted and observed marginals for all features of cells profiled by scRNA-seq of the SciPlex 3 dataset treated with Givinostat. The top 50 marker genes for the perturbation are shown.

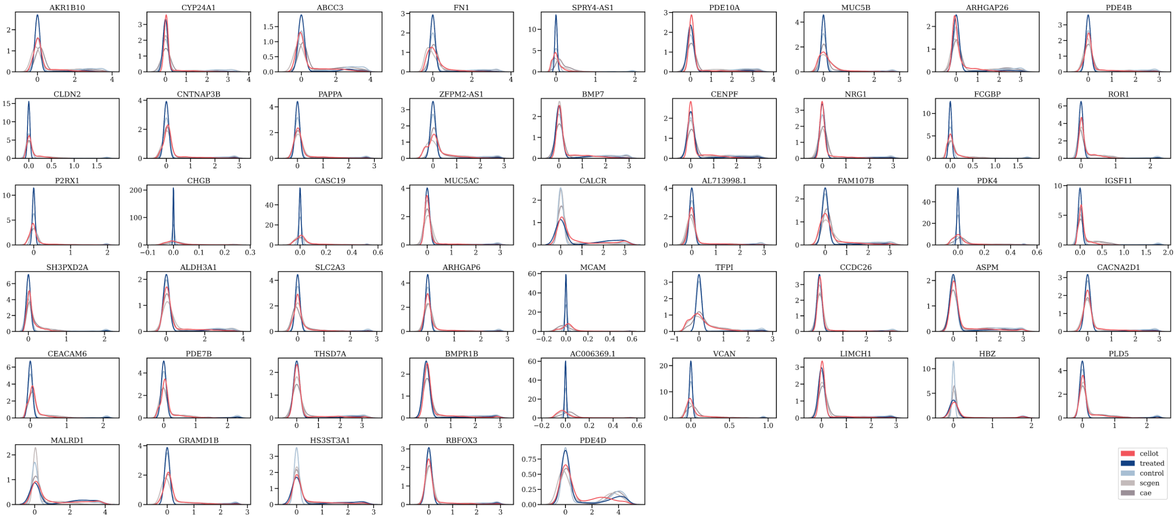

Supplementary Figure 4: Predicted and observed marginals of cells profiled by scRNA-seq of the SciPlex 3 dataset treated with Trametinib. The top 50 marker genes for the perturbation are shown.

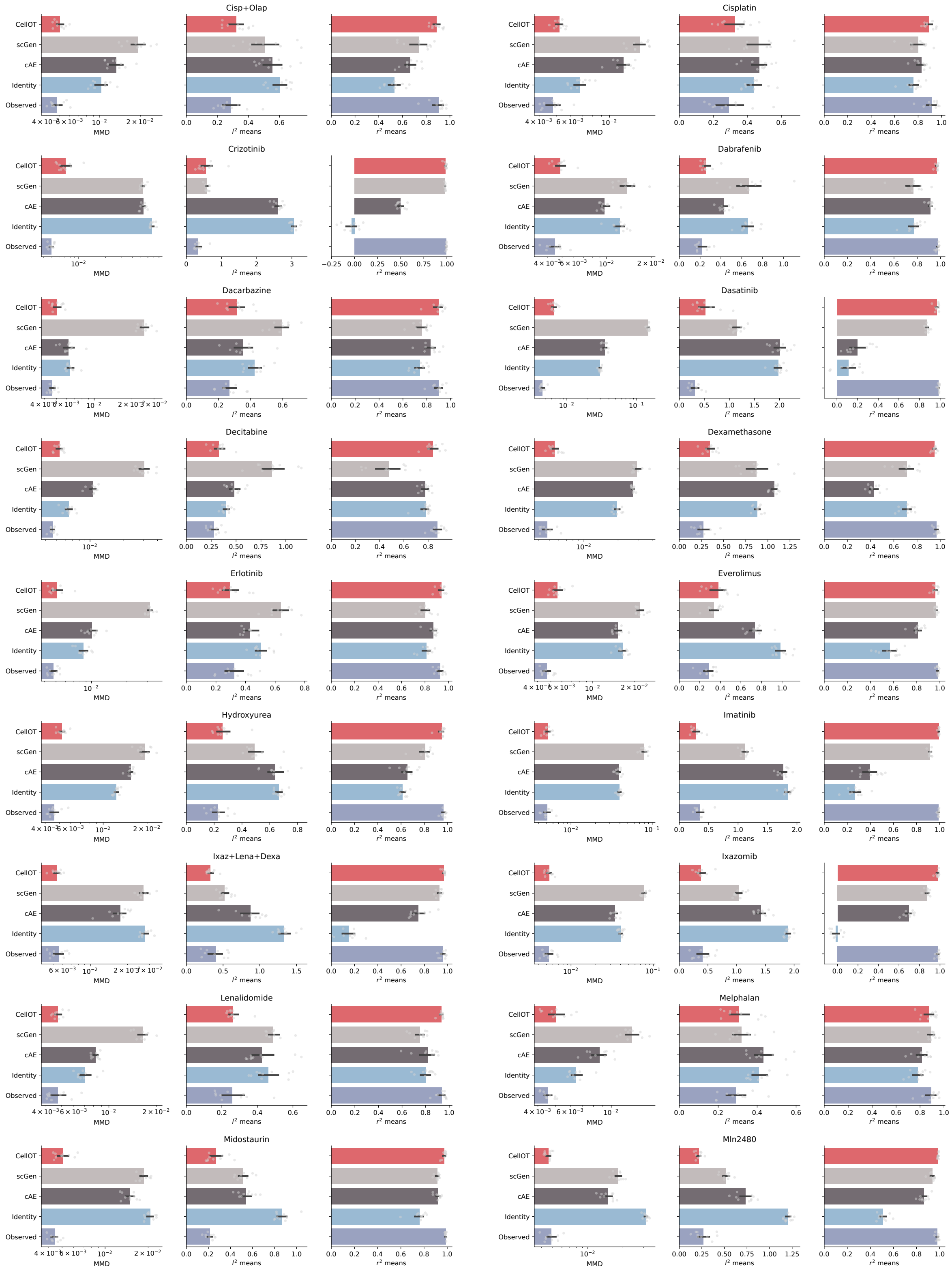

Cisp+Olap
Cisplatin
Crizotinib
Dabrafenib
Dacarbazine
Dasatinib
Decitabine
Dexamethasone
Erlotinib
Everolimus
Hydroxyurea
Imatinib
Ixaz+Lena+Dexa
Ixazomib
Lenalidomide
Melphalan
Midostaurin
Mln2480
CellOT
scGen
cAE
Identity
Observed
MMD
l^2 means
r^2 means

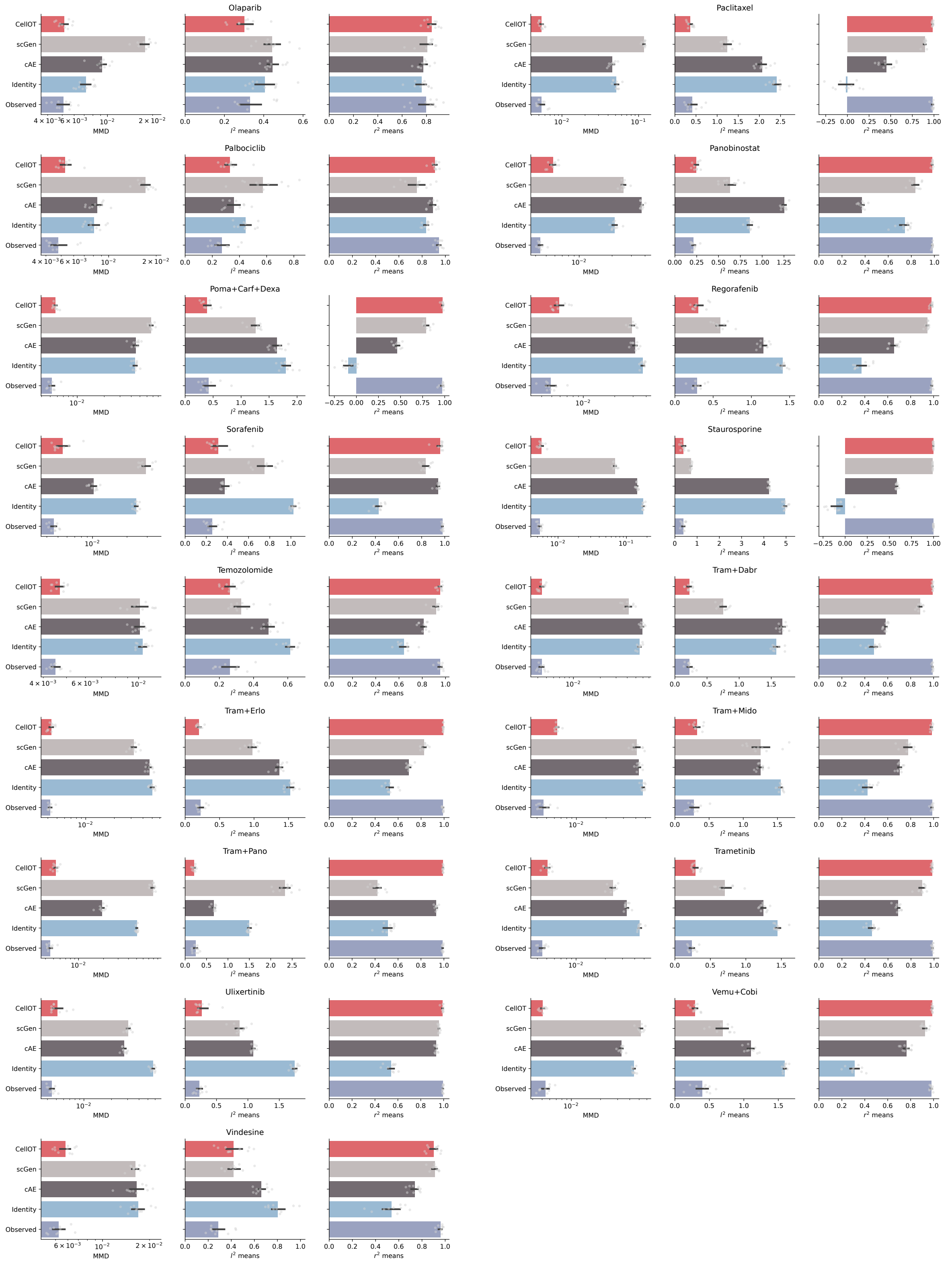

Supplementary Figure 5: Results on other drugs for the 4i dataset for different metrics, including MMD, ℓ_2 feature means, and r^2 correlation feature means for CellOT as well as different baselines. Data is presented as the mean +/- standard deviation across n=10 bootstraps of the test set.

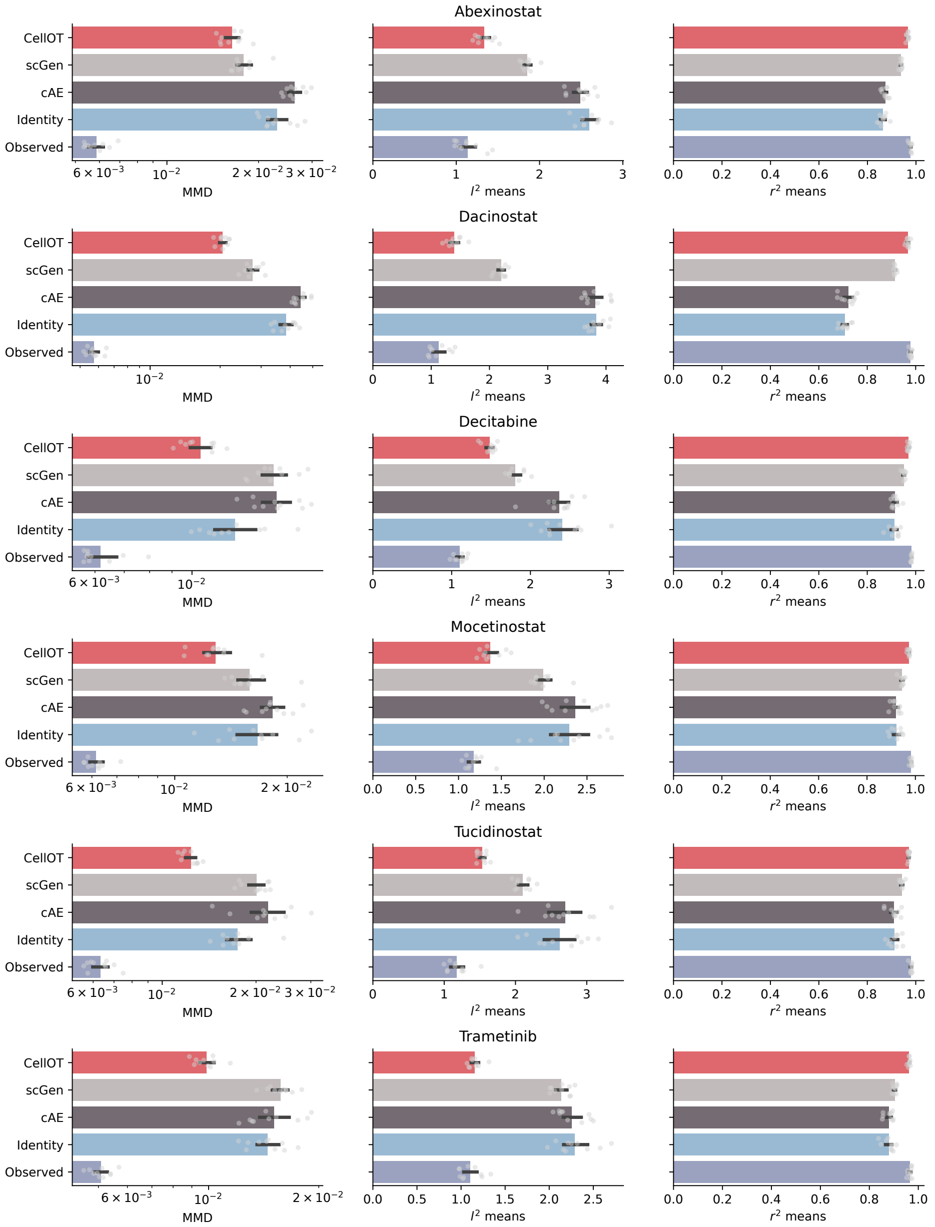

Supplementary Figure 6: Results on other drugs for the SciPlex 3 dataset for different metrics, including MMD, ℓ_2 feature means, and r^2 correlation feature means for CellOT as well as different baselines. Data is presented as the mean +/- standard deviation across n=10 bootstraps of the test set.

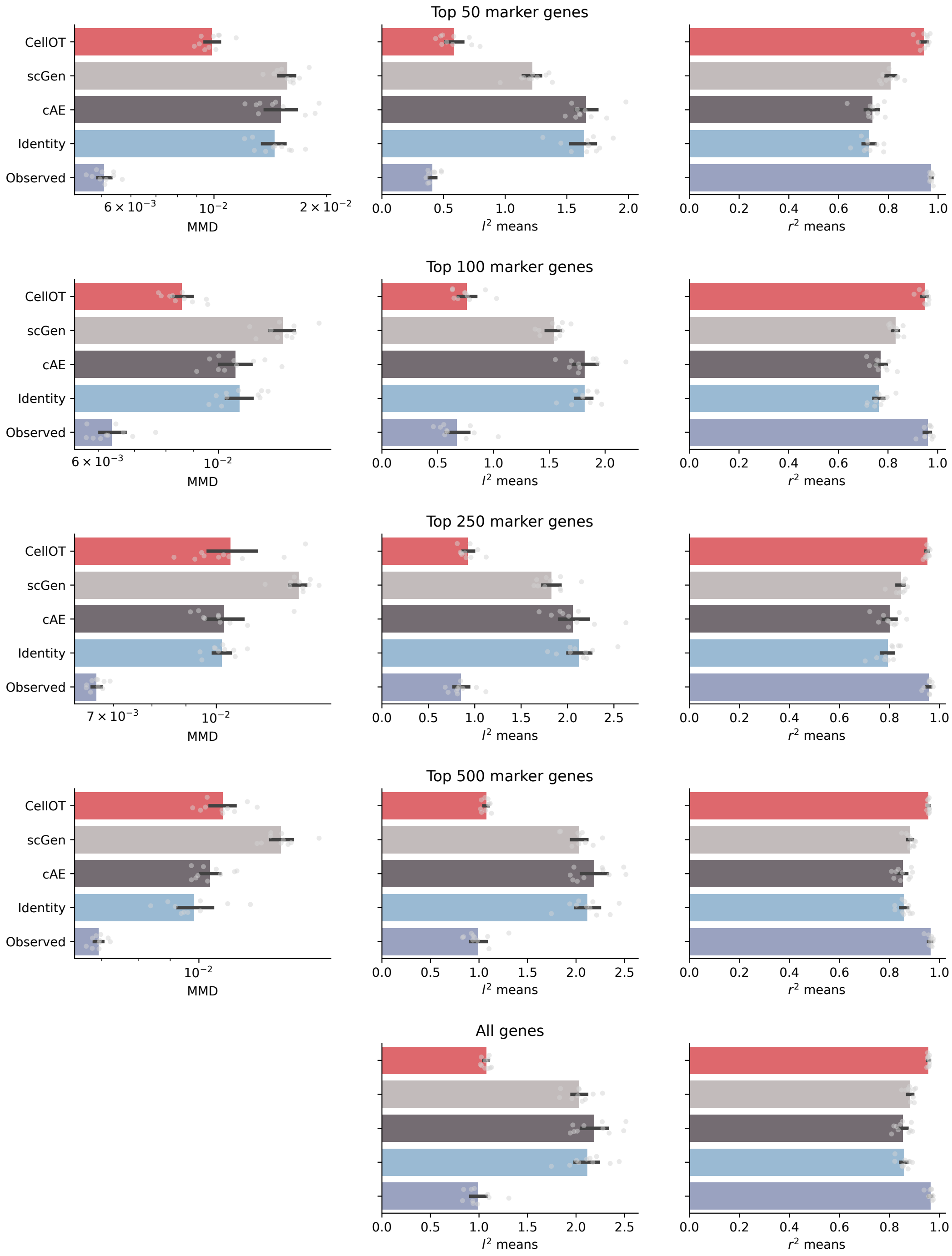

Supplementary Figure 7: Results for single-cell responses for Trametinib the SciPlex 3 dataset for different metrics computed on 50, 100, 250, and 500 marker genes, including MMD, ℓ_2 feature means, and r^2 correlation feature means for CellOT as well as different baselines. With increasing dimensionality, the MMD computation is biased. Data is presented as the mean +/- standard deviation across n=10 bootstraps of the test set.

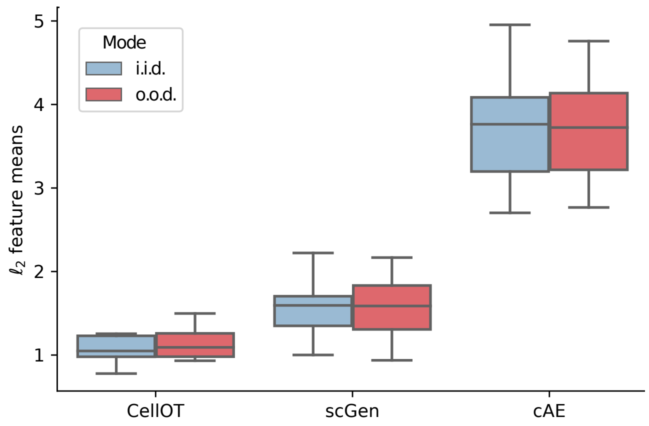

Supplementary Figure 8: ℓ_2 feature means between the predicted distribution and the observed treated distribution for the lupus patients dataset across all holdout samples in the i.i.d. and o.o.s. settings. Boxplots show the median and quartiles of the distribution for 10x bootstraps for each of the n=8 samples.

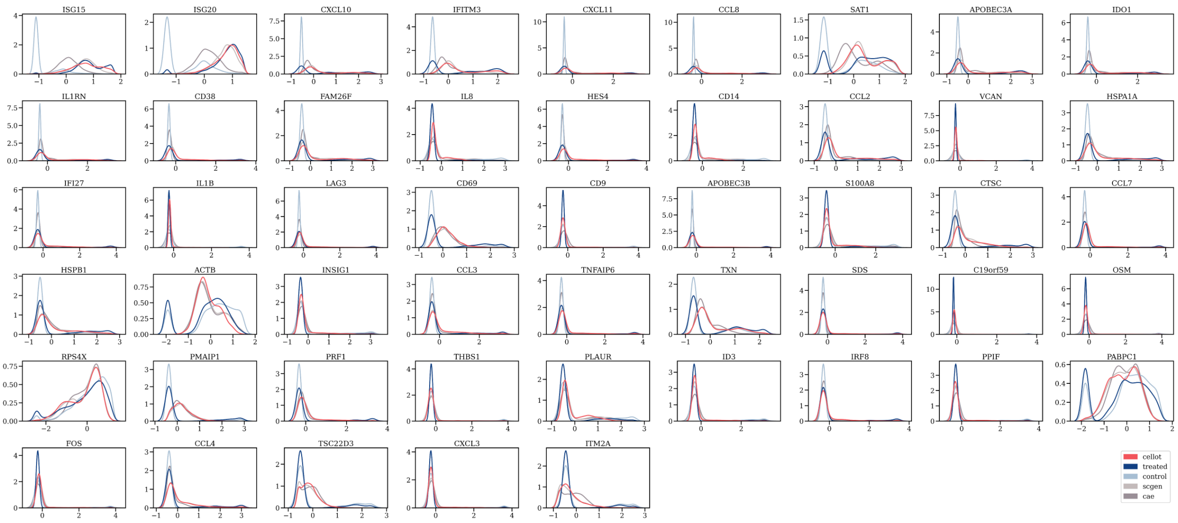

Supplementary Figure 9: Complete set of predicted marginals for scRNA-seq profiled cells of holdout cells pooled across all lupus patients.

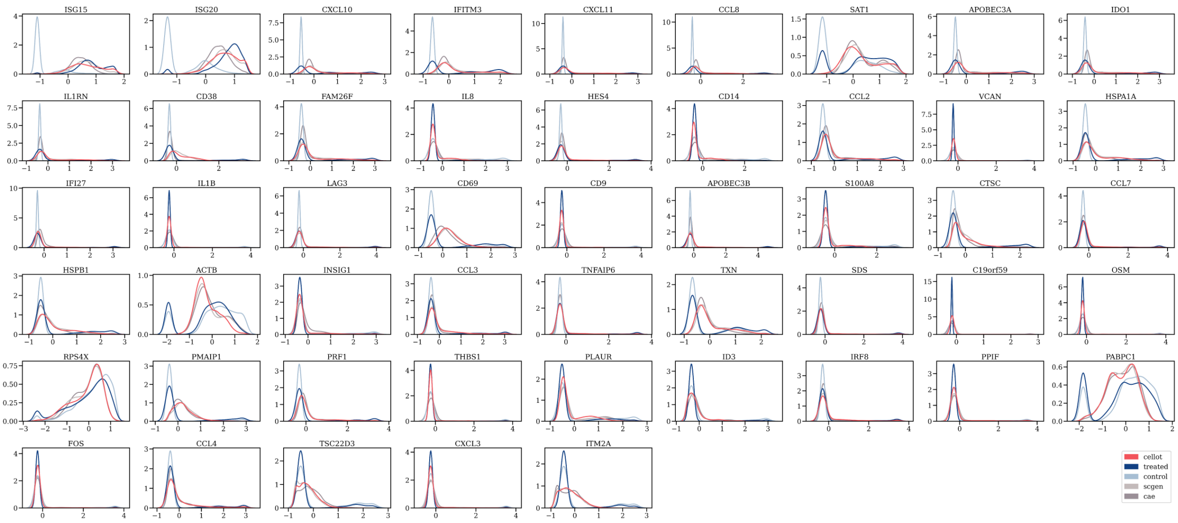

Supplementary Figure 10: Complete set of predicted marginals of scRNA-seq profiled cells from a single holdout lupus patient (id=1015), treated with an IFN-β perturbation.

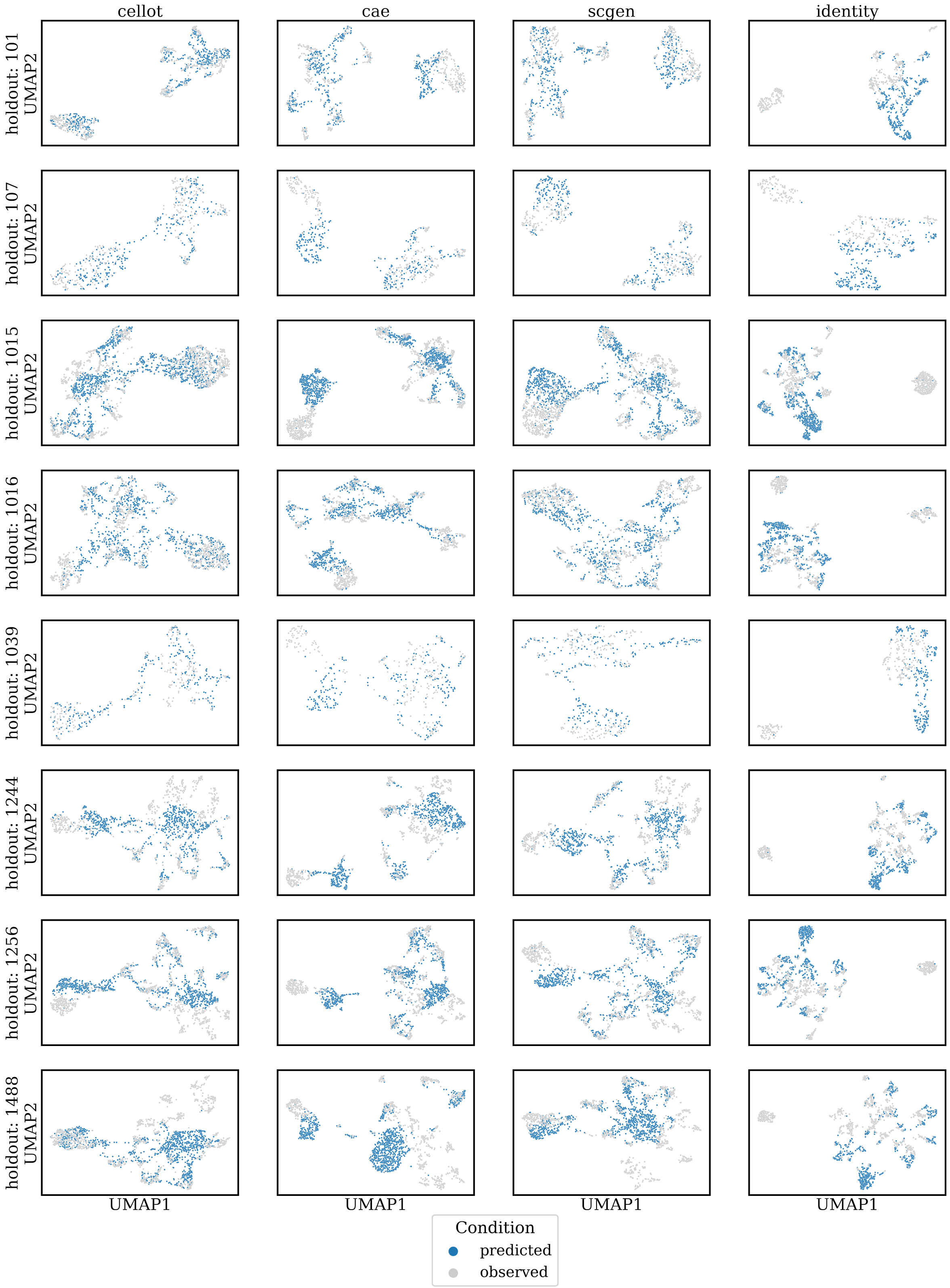

Supplementary Figure 11: UMAP projections of CellOT, different baselines, and naïve OT maps for predicting patient responses to IFN-β treatment for different lupus patients taken as holdout (in the o.o.d. setting). For each method and setting, we display the measured perturbed and predicted perturbed cells.

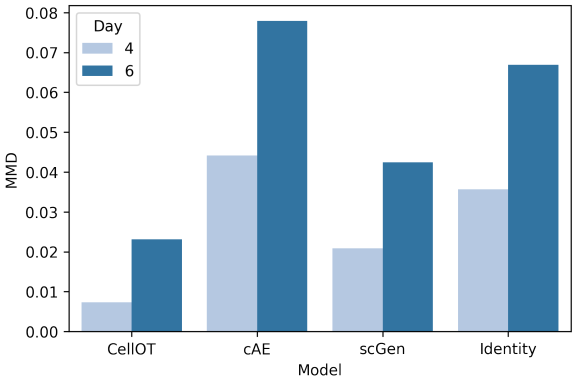

Supplementary Figure 12: Performance w.r.t. the MMD metric between measured perturbed and predicted perturbed cells by CellOT, different baselines, and naiv̈e OT maps on predicting cell differentiation of the statefate data over 4 and 6 days, respectively.

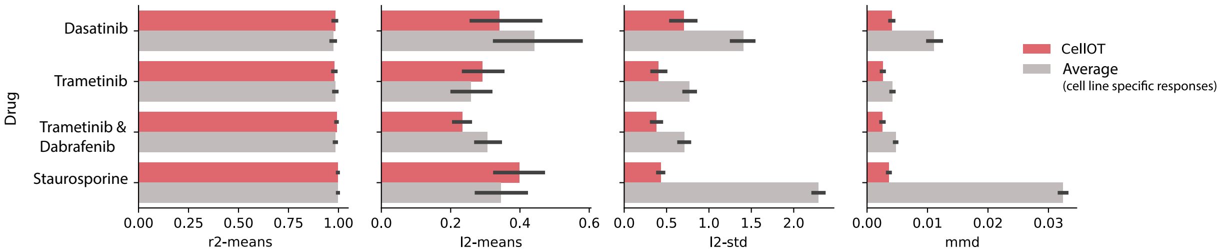

Supplementary Figure 13: Results of predicting perturbation effects of a selection of cancer drugs on 4i data using CellOT and a baseline that predict the average perturbation effect of each cell line (Average). Contrary to CellOT, the baseline requires cell typing, and annotation might not always be trivial. For example, in this setting, cell type markers are affected by the perturbation itself. The benchmark is conducted w.r.t. different metrics, including MMD, r^2 correlation feature means, ℓ_2 feature means, and standard deviation. Data are presented as the mean +/- standard deviation across n=15 bootstraps of the test set.

Supplementary Table 1: Hyperparameter search for scRNA autoencoders.

Parameter	Values	Selected
latent dimension	50, 100	100
num layers	2, 3	2
layer width	256, 512	512
dropout rate	0, 0.05, 0.1, 0.2	0
weight decay	0, 1e-5, 1e-3	1e-5
scheduler.step_size	10k, 50k, 100k	100k
scheduler.gamma	0.1, 0.25, 0.5, 0.9	0.5

C Materials

Cell lines and cell culture media Cell lines M130219 and M130429 are derived from the same human patient suffering from Melanoma cancer. M130219 originates from a subcutaneous biopsy, whereas M130429 originates from a bone biopsy. Cells were tested for the absence of mycoplasm before use and were gifted from the Levesque lab (University of Zürich/ University Hospital Zürich). Culture medium (CM) consists of 10% heat-inactivated Fetal Calf Serum (FCS), 1% Sodium Pyruvate, and 5% Glutamine in RPMI with 0.1mg/ml Anti/Anti. RPMI

without L-Glutamine (Sigma Aldrich), Fetal Calf Serum (Gibco), Sodium Pyruvate (Gibco), Glutamine (Biochrome), Anti/Anti (Gibco).

Pharmacological perturbations For a complete list of compounds, manufacturers and concentrations see Supplementary Table S2. In general, compounds were stored at 5mM in dimethyl sulfoxide (DMSO) and diluted in three steps in CM to 5μM (0.5% DMSO) immediately before use on the cells. In the case of compound combinations, the final concentration of individual compounds was 5μM in CM (and 0.5% DMSO). (Aldrich Material ID: S990051-EA).

4i blocking solution (sBS) sBS consists of 1% Bovine Serum Albumine (BSA), and 150mM Maleimide in phosphate-buffered saline (PBS). Maleimide is added to the aqueous solution just before Blocking step in 4i protocol. BSA (Sigma Aldrich), Maleimide (Sigma Aldrich).

Conventional blocking solution (cBS) cBS consists of 1% Bovine Serum Albumine (BSA) (Sigma Aldrich) in phosphate-buffered saline (PBS).

Imaging buffer (IB) IB consists of 700mM N-Acetyl-Cysteine (NAC) in deionized water (dH20) and 0.1M 4-(2-hydroxyethyl)-1-piperazineethanesulfonic acid (HEPES). Adjust to pH 7.4. NAC (Sigma Aldrich), HEPES (Sigma Aldrich).

Elution buffer (EB) EB consists of 0.5M L-Glycine, 1.2M Urea, 3M Guanidinum chloride (GC), and 70mM TCEP-HCl (TCEP) in dH20. Adjust to pH 2.5. L-Glycine (Sigma-Aldrich), Urea (Sigma-Aldrich), GC (Sigma-Aldrich), TCEP (Sigma-Aldrich).

DNA stain 4', 6-Diamidino-2-phenylindole (DAPI) at 0.4μg/mL in cBS. DAPI (Lifetechnologies)

Primary antibodies The primary antibodies are listed below.

#	Name	Manufacturer	Catalogue # (Clone)	Species	Dilution (1/X)	4i Cycle
1	MelA Cocktail	Abcam	ab733	Mouse	400	1
2	Sox9	Abcam	ab185966	Rabbit	1000	1
3	pS6K1	Millipore	MABS82 (10G7.1)	Mouse	800	2
4	pAKT	Cell Signaling Technology	4060	Rabbit	600	2
5	PCNA	Abcam	ab139696	Chicken	4000	2
6	pEGFR	Cell Signaling Technology	2236	Mouse	500	3
7	pERK	Cell Signaling Technology	9101	Rabbit	500	3
8	Alpha-Tubulin	Millipore	MAB1864 (YL1/2)	Rat	8000	3
9	Ki67	Santa Cruz Biotechnology	sc-23900	Mouse	200	4
10	pMet	Cell Signaling Technology	3077	Rabbit	800	4
11	CD45	Abcam	ab187271	Mouse	400	5
12	Cleaved Caspase-3	Cell Signaling Technology	9664	Rabbit	500	5

Secondary antibodies All secondary antibodies were diluted as listed below with cBS.

#	Name	Manufacturer	Catalogue #	Species	Dilution (1/X)
1	Anti-mouse AlexaFluor-488	Life Technologies (Invitrogen)	A-11029	Goat	400
2	anti-rabbit AlexaFluor-568	Life Technologies (Invitrogen)	A-11036	Goat	400
3	Anti-chicken AlexaFluor- 555	Life Technologies (Invitrogen)	A-32932	Goat	400
4	Anti-rat AlexaFluor- 555	Life Technologies (Invitrogen)	A-21434	Goat	400
5	Anti-rabbit AlexaFluor-647	Life Technologies (Invitrogen)	A-21245	Goat	400

D Experimental details

D.1 In-vitro experiments

Cell culture Cells from both cell lines were cultured in Complete Medium at 37°C, 95% humidity, and 5% CO2. Per well 750 cells of each cell line were seeded in a 384-well plate (Greiner, n°781092, and lid n°656191) and grown for 3 days in the above-mentioned conditions.

Pharmacological perturbations Compounds were added to the cells using the Bravo liquid handling platform (Agilent Technologies) at the concentration specified in the Materials section. Drug perturbations were performed in triplicates (as technical replicates). The cells were then incubated for 8h at 37°C, 95% humidity, and 5% CO2 prior to fixation.

Sample preparation Sample preparation was performed as follows: Cells were fixed in 4% Paraformaldehyde (Electron Microscopy Sciences) for 30min. Cells were then permeabilized with 0.5% Triton X-100 (Manufacturer) for 15 min. Fixation and permeabilization were performed at room temperature.

Iterative indirect immunofluorescence imaging (4i) Each subsequent step was performed in a sequence of mentioning and in every cycle of 4i. If not stated differently, all steps were performed at room temperature. (1) Antibody Elution. The sample was washed 4 times with dH20. Residual dH20 was aspirated to a minimal volume. Subsequent actions are repeated 3 times: EB was added to the sample and shaken at 100 rpm for 10 min. Then EB was aspirated to a minimal volume. (2) Blocking. sBS was added to the sample and shaken at 100 revolutions per minute (rpm) for 1 hour. After 1h sample was washed 3 times with PBS. (3) Indirect immunofluorescence, primary antibody stain. The primary antibody solution was added to the sample and shaken at 100 rpm for 2 hours. After 2 hours, the sample was washed 3 times with PBS. (4) Indirect immunofluorescence, secondary antibody stain. The secondary antibody solution was added to the sample and shaken at 100 rpm for 2 hours. After 2 hours, the sample was washed 3 times with PBS. (5) Imaging. IB was added to the sample and the sample was imaged. Perform steps 1 to 5 until the required plexity is achieved. All liquid dispensing and washing steps of the 4i protocol were performed using a Washer Dispenser EL406 (BioTek). Primary and secondary antibodies were dispensed using a Bravo liquid handling platform (Agilent Technologies).

Nucleus and total cell staining Nuclei were stained using DSS during each 4i cycle by adding DAPI at the above-specified concentration (Materials) to the secondary antibody solution. Between steps 4 and 5 of the last 4i cycles, a cell staining was performed using AlexaFluor-647 NHS Ester (succinimidyl ester) (Invitrogen) for 5 minutes at a final concentration of 0.2μg/mL in 50mM carbonate-bicarbonate buffer pH 9.2. AlexaFluor-647 NHS Ester (Invitrogen, cat#A20006)

Microscopy An automated high-content microscope from GE Healthcare (IN Cell 6000) with an enhanced CSU-W1 spinning disk (Microlens-enhanced dual Nipkow disk confocal scanner, wide view type) was used in combination with a Nikon 40X (0.95 NA), Plan Apo, Correction Collar 0.11-0.23, CFI/Lambda, and Neo sCMOS cameras (Andor, 2,560 × 2,160 pixels) to acquire microscopy images. 7 by 7 images were acquired per well. 7 z-planes with a 1 μm z-spacing were acquired per site and a maximum intensity projection was computed and used for subsequent image analysis. UV (406 nm), green (488 nm), red (568 nm), and far red (625 nm) signals were acquired sequentially.

D.2 In-silico experiments

Image processing Image processing was done using TissueMAPS (TM): a cloud-based, interactive image processing and viewing tool developed by the Pelkmans Lab (`https://`

github.com/TissueMAPS). As the first step during image processing, images were corrected for an illumination bias (108). Next, corrected images from different acquisition cycles from the same microscopy site were aligned as previously described (109). Finally, corrected and aligned images were used to generate pyramid views of the entire dataset, which were later used to train classifiers (see below).

Image analysis and feature extraction Image analysis and feature extraction were performed using TM. Nuclei were segmented using DAPI signal of the first 4i cycle by applying Otsu thresholding and morphologically filling the identified objects (TM jterator modules: threshold_otsu & separate_clumps). Cell segmentation was performed using the AlexaFluor-647 NHS Ester (Sucs) signal acquired during the last 4i cycle by smoothing the Sucs signal, and adaptive thresholding (TM jterator modules: smooth & segment_secondary). Nucleus and cell morphology features were measured using TM jterator module measure_morphology. Prior to intensity feature extraction, all images were corrected for background signal by subtracting 120 pixel values from each pixel (TM jterator module: rescale). Intensity features were extracted for nucleus and cell objects using TM jterator module measure_intensity. The cell data extracted from drug treatment replicates was consolidated under the share drug label, replicate information was not further used.

Semi-supervised classifiers and data clean-up Cells tainted by artifacts related to sample preparation and image analysis (e.g., miss-segmentation, detachment during 4i procedure, fluorescent debris) were manually selected using TM's graphical interface and used to train random forest classifiers to systematically exclude cells with similar artifacts from the dataset. Further, cells whose segmentation masks touched image boundaries were also excluded from the dataset.

Identification of cell states Single-cell intensity and morphology features of DMSO-treated (control) cells, for which perturbation effects were predicted using CellOT, were clustered using the Leiden algorithm (110) provided by the Python package `scanpy` (78, `scanpy.tl.leiden`) without customization of input parameters. Prior to Leiden clustering, a neighborhood graph was constructed for the Control cells using `scanpy.pp.neighbors` with the input parameter `_neighbors` = 10 (no further customization of the input parameters).

UMAP generation Uniform Manifold Approximation and Projection visualizations in Fig. 2, 3, and Supplementary Figure 11, and Extended Data Figure 5 were generated using `scanpy`'s `scanpy.pl.umap` function preceded by `scanpy.pp.neighbors` (111).

3NN cell measurement The three nearest neighbor cells measurement (3NN) was calculated by identifying the three nearest cells of either measured or predicted cells in the population of measured cells using all features except pERK and then averaging their pERK value. The nearest neighbor search was performed for each drug condition separately.

Prediction tasks in the i.i.d. setting All marginals, UMAPs, and metrics in Fig. 2 and Supplementary Figure 5 and Supplementary Figure 6 are computed using the unseen test set cells. UMAP projections are computed on the joint set of predicted and measured cells. The larger set is down samples such that their sizes are equal.

Prediction tasks in the o.o.s. and o.o.d. setting We test the ability of CellOT to generalize to out-of-sample (o.o.s.) and out-of-distribution (o.o.d.) settings by predicting perturbation response on holdout samples and development trajectory on holdout cellular subpopulations. Results are reported in Fig. 4, Supplementary Figure 8, Extended Data Figure 4, and Extended Data Figure 6. To measure the drop in performance when switching to the o.o.d. or o.o.s. setting, for each holdout group, two models are trained, an i.i.d. and

o.o.s./o.o.d. model. Both models are trained on all cells from the other groups, however, the i.i.d. model is additionally trained on half of the cells of the holdout group. Evaluations for both settings are done using the cells unseen by the i.i.d. model.

References

[82] Bo Yuan, Ciyue Shen, Augustin Luna, Anil Korkut, Debora S Marks, John Ingraham, and Chris Sander. CellBox: Interpretable Machine Learning forPerturbation Biology with Application to the Designof Cancer Combination Therapy. *Cell Systems*, 12(2), 2021.

[83] Fabian Fröhlich, Thomas Kessler, Daniel Weindl, Alexey Shadrin, Leonard Schmiester, Hendrik Hache, Artur Muradyan, Moritz Schütte, Ji-Hyun Lim, Matthias Heinig, et al. Efficient parameter estimation enables the prediction of drug response using a mechanistic pan-cancer pathway model. *Cell Systems*, 7(6):567–579, 2018.

[84] Berend Snijder, Raphael Sacher, Pauli Rämö, Prisca Liberali, Karin Mench, Nina Wolfrum, Laura Burleigh, Cameron C Scott, Monique H Verheije, Jason Mercer, et al. Single-cell analysis of population context advances RNAi screening at multiple levels. *Molecular Systems Biology*, 8(1):579, 2012.

[85] Doris Berchtold, Nico Battich, and Lucas Pelkmans. A systems-level study reveals regulators of membrane-less organelles in human cells. *Molecular Cell*, 72(6), 2018.

[86] Victoria A Green and Lucas Pelkmans. A systems survey of progressive host-cell reorganization during rotavirus infection. *Cell Host & Microbe*, 20(1):107–120, 2016.

[87] Atray Dixit, Oren Parnas, Biyu Li, Jenny Chen, Charles P Fulco, Livnat Jerby-Arnon, Nemanja D Marjanovic, Danielle Dionne, Tyler Burks, Raktima Raychowdhury, et al. Perturb-Seq: Dissecting Molecular Circuits with Scalable Single-Cell RNA Profiling of Pooled Genetic Screens. *Cell*, 167(7):1853–1866, 2016.

[88] Kenji Kamimoto, Christy M Hoffmann, and Samantha A Morris. CellOracle: Dissecting cell identity via network inference and in silico gene perturbation. *bioRxiv*, 2020.

[89] Tiam Heydari, Matthew A. Langley, Cynthia L Fisher, Daniel Aguilar-Hidalgo, Shreya Shukla, Ayako Yachie-Kinoshita, Michael Hughes, Kelly M. McNagny, and Peter W Zandstra. IQCELL: A platform for predicting the effect of gene perturbations on developmental trajectories using single-cell RNA-seq data. *PLoS Computational Biology*, 18(2), 2022.

[90] Romain Lopez, Jeffrey Regier, Michael B Cole, Michael I Jordan, and Nir Yosef. Deep generative modeling for single-cell transcriptomics. *Nature Methods*, 15(12):1053–1058, 2018.

[91] Mohammad Lotfollahi, F Alexander Wolf, and Fabian J Theis. scGen predicts single-cell perturbation responses. *Nature Methods*, 16(8), 2019.

[92] Sisi Chen, Paul Rivaud, Jong H Park, Tiffany Tsou, Emeric Charles, John R Haliburton, Flavia Pichiorri, and Matt Thomson. Dissecting heterogeneous cell populations across drug and disease conditions with PopAlign. *Proceedings of the National Academy of Sciences*, 117(46), 2020.

[93] Megasthenis Asteris, Dimitris Papailiopoulos, and Alexandros G Dimakis. Orthogonal NMF through Subspace Exploration. In *Advances in Neural Information Processing Systems (NeurIPS)*, volume 28, 2015.

[94] William S Chen, Nevena Zivanovic, David Van Dijk, Guy Wolf, Bernd Bodenmiller, and Smita Krishnaswamy. Uncovering axes of variation among single-cell cancer specimens. *Nature Methods*, 17(3), 2020.

[95] Sherry Bhalla, David T Melnekoff, Adolfo Aleman, Violetta Leshchenko, Paula Restrepo, Jonathan Keats, Kenan Onel, Jeffrey R Sawyer, Deepu Madduri, Joshua Richter, et al. Patient similarity network of newly diagnosed multiple myeloma identifies patient subgroups with distinct genetic features and clinical implications. *Science Advances*, 7(47), 2021.

[96] Tara Chari, Brandon Weissbourd, Jase Gehring, Anna Ferraioli, Lucas Leclère, Makenna Herl, Fan Gao, Sandra Chevalier, Richard R Copley, Evelyn Houliston, et al. Whole-animal multiplexed single-cell RNA-seq reveals transcriptional shifts across Clytia medusa cell types. *Science Advances*, 7(48), 2021.

[97] Michael A Skinnider, Jordan W Squair, Claudia Kathe, Mark A Anderson, Matthieu Gautier, Kaya JE Matson, Marco Milano, Thomas H Hutson, Quentin Barraud, Aaron A Phillips, et al. Cell type prioritization in single-cell data. *Nature Biotechnology*, 39(1), 2021.

[98] Daniel B Burkhardt, Jay S Stanley, Alexander Tong, Ana Luisa Perdigoto, Scott A Gigante, Kevan C Herold, Guy Wolf, Antonio J Giraldez, David van Dijk, and Smita Krishnaswamy. Quantifying the effect of experimental perturbations at single-cell resolution. *Nature Biotechnology*, 39(5), 2021.

[99] Viktor Petukhov, Anna A Igolkina, Rasmus Rydbirk, Shenglin Mei, Lars Christoffersen, Konstantin Khodosevich, and Peter Kharchenko. Case-control analysis of single-cell RNA-seq studies. *bioRxiv*, 2022.

[100] Yue Cao, Pengyi Yang, and Jean Yee Hwa Yang. A benchmark study of simulation methods for single-cell rna sequencing data. *Nature Communications*, 12(1), 2021.

[101] Geoffrey Schiebinger, Jian Shu, Marcin Tabaka, Brian Cleary, Vidya Subramanian, Aryeh Solomon, Joshua Gould, Siyan Liu, Stacie Lin, Peter Berube, et al. Optimal-Transport Analysis of Single-Cell Gene Expression Identifies Developmental Trajectories in Reprogramming. *Cell*, 176(4), 2019.

[102] Hugo Lavenant, Stephen Zhang, Young-Heon Kim, and Geoffrey Schiebinger. Towards a mathematical theory of trajectory inference. *arXiv preprint arXiv:2102.09204*, 2021.

[103] Leygonie Jacob, Jennifer She, Amjad Almahairi, Sai Rajeswar, and Aaron Courville. W2GAN: Recovering an Optimal Transport Map with a GAN. *arXiv Preprint*, 2018.

[104] Karren D Yang and Caroline Uhler. Scalable Unbalanced Optimal Transport using Generative Adversarial Networks. In *International Conference on Learning Representations (ICLR)*, 2019.

[105] Neha Prasad, Karren Yang, and Caroline Uhler. Optimal Transport using GANs for Lineage Tracing. *arXiv preprint arXiv:2007.12098*, 2020.

[106] Ashok Makkuva, Amirhossein Taghvaei, Sewoong Oh, and Jason Lee. Optimal transport mapping via input convex neural networks. In *International Conference on Machine Learning (ICML)*, volume 37, 2020.

[107] Brandon Amos, Lei Xu, and J Zico Kolter. Input Convex Neural Networks. In *International Conference on Machine Learning (ICML)*, volume 34, 2017.

[108] Nico Battich, Thomas Stoeger, and Lucas Pelkmans. Image-based transcriptomics in thousands of single human cells at single-molecule resolution. *Nature Methods*, 10(11), 2013.

[109] Gabriele Gut, Markus D Herrmann, and Lucas Pelkmans. Multiplexed protein maps link subcellular organization to cellular states. *Science*, 361(6401), 2018.

[110] Vincent A Traag, Ludo Waltman, and Nees Jan Van Eck. From Louvain to Leiden: guaranteeing well-connected communities. *Scientific Reports*, 9(1), 2019.

[111] F Alexander Wolf, Philipp Angerer, and Fabian J Theis. SCANPY: large-scale single-cell gene expression data analysis. *Genome Biology*, 19(1), 2018.
