# Supplementary material for: Learning single-cell perturbation responses using neural optimal transport
Source: Nat Methods. 2023 Sep 28;20(11):1759–68. doi: 10.1038/s41592-023-01969-x (PMC10630137; doi:10.1038/s41592-023-01969-x)
Supplement: Supplementary file 2 — Reporting Summary [file 41592_2023_1969_MOESM2_ESM.pdf]

nature research

Corresponding author(s): Rätsch (and Krause, Pelkmans, Lehmann)

Last updated by author(s): Apr 27, 2023

# Reporting Summary

Nature Research wishes to improve the reproducibility of the work that we publish. This form provides structure for consistency and transparency in reporting. For further information on Nature Research policies, see our Editorial Policies and the Editorial Policy Checklist.

## Statistics

For all statistical analyses, confirm that the following items are present in the figure legend, table legend, main text, or Methods section.

| n/a | Confirmed | |
|---|---|---|
| ☒ | ☐ | The exact sample size (*n*) for each experimental group/condition, given as a discrete number and unit of measurement |
| ☒ | ☐ | A statement on whether measurements were taken from distinct samples or whether the same sample was measured repeatedly |
| ☒ | ☐ | The statistical test(s) used AND whether they are one- or two-sided<br>*Only common tests should be described solely by name; describe more complex techniques in the Methods section.* |
| ☒ | ☐ | A description of all covariates tested |
| ☒ | ☐ | A description of any assumptions or corrections, such as tests of normality and adjustment for multiple comparisons |
| ☒ | ☐ | A full description of the statistical parameters including central tendency (e.g. means) or other basic estimates (e.g. regression coefficient) AND variation (e.g. standard deviation) or associated estimates of uncertainty (e.g. confidence intervals) |
| ☒ | ☐ | For null hypothesis testing, the test statistic (e.g. *F*, *t*, *r*) with confidence intervals, effect sizes, degrees of freedom and *P* value noted<br>*Give P values as exact values whenever suitable.* |
| ☒ | ☐ | For Bayesian analysis, information on the choice of priors and Markov chain Monte Carlo settings |
| ☒ | ☐ | For hierarchical and complex designs, identification of the appropriate level for tests and full reporting of outcomes |
| ☒ | ☐ | Estimates of effect sizes (e.g. Cohen's *d*, Pearson's *r*), indicating how they were calculated |

*Our web collection on statistics for biologists contains articles on many of the points above.*

## Software and code

Policy information about availability of computer code

| | |
|---|---|
| Data collection | A detailed description of the 4i related software that was used to collect marker and other cell associated information is described in the Online Methods section. Moreover, the preprocessing of the scRNA data is also described in the Online Methods section. |
| Data analysis | This work presents a new analysis method, described in detail in the Online Methods section. The code is available online (see Code Availability statement). Additional details on in-vitro and in-silico experiments can be found in Section S4 of the Supplementary Information. |

For manuscripts utilizing custom algorithms or software that are central to the research but not yet described in published literature, software must be made available to editors and reviewers. We strongly encourage code deposition in a community repository (e.g. GitHub). See the Nature Research guidelines for submitting code & software for further information.

## Data

Policy information about availability of data

All manuscripts must include a data availability statement. This statement should provide the following information, where applicable:

- Accession codes, unique identifiers, or web links for publicly available datasets
- A list of figures that have associated raw data
- A description of any restrictions on data availability

Raw published data for the SciPlex 3 (31), lupus patients (38), glioblastoma patients (44), and statefate dataset (46) are available from the Gene Expression Omnibus under accession codes GSM4150378, GSE96583, GSE148842, and GSE140802, respectively. Data from the cross species dataset (45) is hosted on the BioStudies database of EMBL-EBI under code E-MTAB-6754. A full set of links can be found in their publication. Details on web links for raw 4i melanoma data and all processed datasets can be found in the Data Availability section.

# Field-specific reporting

Please select the one below that is the best fit for your research. If you are not sure, read the appropriate sections before making your selection.

☒ Life sciences ☐ Behavioural & social sciences ☐ Ecological, evolutionary & environmental sciences

For a reference copy of the document with all sections, see nature.com/documents/nr-reporting-summary-flat.pdf

# Life sciences study design

All studies must disclose on these points even when the disclosure is negative.

**Sample size**
We use two cell lines and treated them with 34 drugs and combinations of drugs. Each experiment contains thousands of cells that were profiled with the 4i technology.

**Data exclusions**
Details in Section S4.2 of the Supplement. Specifically, about the exclusion of cells in the subsequent analysis, we state: "Cells tainted by artifacts related to sample preparation and image analysis (e.g., miss-segmentation, detachment during 4i procedure, fluorescent debris) were manually selected using TM's graphical interface and used to train random forest classifiers to systematically exclude cells with similar artifacts from the dataset. Further, cells whose segmentation masks touched image boundaries were also excluded from the dataset."

**Replication**
*Describe the measures taken to verify the reproducibility of the experimental findings. If all attempts at replication were successful, confirm this OR if there are any findings that were not replicated or cannot be reproduced, note this and describe why.*

**Randomization**
We split the data into training and test set for training and evaluating the method. The Online Methods contains a more detailed description of the setup and also how we consider the out of sample and out of distribution settings.

**Blinding**
Blinding was not relevant.

# Reporting for specific materials, systems and methods

We require information from authors about some types of materials, experimental systems and methods used in many studies. Here, indicate whether each material, system or method listed is relevant to your study. If you are not sure if a list item applies to your research, read the appropriate section before selecting a response.

## Materials & experimental systems

| n/a | Involved in the study | |
|---|---|---|
| ☐ | ☒ | Antibodies |
| ☐ | ☒ | Eukaryotic cell lines |
| ☒ | ☐ | Palaeontology and archaeology |
| ☒ | ☐ | Animals and other organisms |
| ☒ | ☐ | Human research participants |
| ☒ | ☐ | Clinical data |
| ☒ | ☐ | Dual use research of concern |

## Methods

| n/a | Involved in the study | |
|---|---|---|
| ☒ | ☐ | ChIP-seq |
| ☒ | ☐ | Flow cytometry |
| ☒ | ☐ | MRI-based neuroimaging |

## Antibodies

**Antibodies used**
A detailed table of antibodies is provided in Section S3 of the Supplements.

**Validation**
*Describe the validation of each primary antibody for the species and application, noting any validation statements on the manufacturer's website, relevant citations, antibody profiles in online databases, or data provided in the manuscript.*

## Eukaryotic cell lines

Policy information about cell lines

**Cell line source(s)**
We consider two co-cultured primary melanoma cell lines (M130219 and M130429), which were derived from the same melanoma patient from different body sites. M130219 originates from a subcutaneous biopsy taken during treatment with Bimetinib (MEKi), whereas M130429 was derived from a bone autopsy one month after stopping said targeted therapy (29). Described first here: https://doi.org/10.1111/exd.12683

**Authentication**
The cell lines were gifted from the Mitchell Levesque (University of Zürich/ University Hospital Zürich) senior author of publication that first described the cell lines and not explicitly authenticated.

| | |
|---|---|
| Mycoplasma contamination | Cells were tested for the absence of mycoplasm before use. |
| Commonly misidentified lines<br>(See ICLAC register) | none |
